# Supplementary material for: Quantifying evolutionary constraints on B-cell affinity maturation
Source: Philos Trans R Soc Lond B Biol Sci. 2015 Sep 5;370(1676):20140244. doi: 10.1098/rstb.2014.0244 (PMC4528421; doi:10.1098/rstb.2014.0244)
Supplement: Supplementary Material [file rstb20140244supp1.pdf]

Supplementary Material: Quantifying evolutionary constraints on B cell affinity maturation

CONNOR O. MCCOY, TREVOR BEDFORD, VLADIMIR N. MININ, PHILIP BRADLEY, HARLAN ROBINS, AND FREDERICK A. MATSEN IV
Philosophical Transactions of the Royal Society B; doi:10.1098/rstb.2014-0244

SUPPLEMENTARY METHODS

Derivation of the Gamma-Poisson marginal likelihood with varying observation depth. We will use the same notation as in the Materials and Methods section. Our first task is to write down a likelihood of α and β given a collection of counts. To do so we will marginalize out the rates λ_l when they are drawn from a $\mathrm{Gamma}(\alpha, \beta)$ as in the main text.

The likelihood for a single site is (omitting l for now):

$$\begin{aligned} P(C|t,\alpha,\beta) &= \int_0^\infty P(C|t,\lambda)P(\lambda|\alpha,\beta)d\lambda \\ &= \int_0^\infty \frac{(\lambda t)^C e^{-\lambda t}}{C!} P(\lambda|\alpha,\beta)d\lambda \\ &= \int_0^\infty \frac{(\lambda t)^C e^{-\lambda t}}{C!} \left[\frac{\beta^\alpha}{\Gamma(\alpha)}\lambda^{\alpha-1}e^{-\beta\lambda}\right] d\lambda \\ &= \frac{\beta^\alpha t^C}{C!\,\Gamma(\alpha)} \int_0^\infty \lambda^{C+\alpha-1} e^{-\lambda(t+\beta)} d\lambda. \end{aligned}$$

Letting $\alpha' = C + \alpha$ and $\beta' = t + \beta$, introduce a normalizing constant for the distribution $\mathrm{Gamma}(\alpha', \beta')$:

$$\begin{aligned} P(C|t,\alpha,\beta) &= \frac{\beta^\alpha t^C}{C!\,\Gamma(\alpha)} \frac{\Gamma(\alpha')}{\beta'^{\alpha'}} \int_0^\infty \frac{\beta'^{\alpha'}}{\Gamma(\alpha')} \lambda^{\alpha'-1} e^{-\lambda(\beta')} d\lambda \\ &= \frac{\beta^\alpha t^C}{C!\,\Gamma(\alpha)} \frac{\Gamma(\alpha')}{\beta'^{\alpha'}} \int_0^\infty \mathrm{DGamma}(\lambda;\alpha',\beta') d\lambda. \end{aligned}$$

The integral over the support of the Gamma distribution is 1, so:

$$\begin{aligned} P(C|t,\alpha,\beta) &= \frac{\beta^\alpha t^C}{C!\,\Gamma(\alpha)} \frac{\Gamma(\alpha')}{\beta'^{\alpha'}} \\ &= \frac{\beta^\alpha t^C}{C!\,\Gamma(\alpha)} \frac{\Gamma(C+\alpha)}{(t+\beta)^{C+\alpha}}. \end{aligned}$$

The overall marginal likelihood is the product over such sites:

$$\begin{aligned}\mathcal{L} &= P(C_1,\dots,C_L|t_1,\dots,t_L,\alpha,\beta) = \prod_l \frac{\beta^\alpha t_l^{C_l}}{C_l!\,\Gamma(\alpha)} \frac{\Gamma(C_l+\alpha)}{(t_l+\beta)^{C_l+\alpha}} \\ &= \left(\frac{\beta^\alpha}{\Gamma(\alpha)}\right)^L \prod_l \frac{t_l^{C_l}}{C_l!} \frac{\Gamma(C_l+\alpha)}{(t_l+\beta)^{C_l+\alpha}} \\ &= \left(\frac{\beta^\alpha}{\Gamma(\alpha)}\right)^L \prod_l \frac{t_l^{C_l}}{\Gamma(C_l+1)} \frac{\Gamma(C_l+\alpha)}{(t_l+\beta)^{C_l+\alpha}},\end{aligned}$$

giving (1).

Posterior for λ. Our eventual goal is a regularized posterior estimate of the rates λ_l. For a single site, once again dropping l:

$$P(\lambda|C,t,\hat\alpha,\hat\beta) \propto P(C|\lambda,t)P(\lambda|\hat\alpha,\hat\beta).$$

Substituting in the PDFs for the distributions employed for C and λ:

$$P(\lambda|C,t,\hat\alpha,\hat\beta) \propto \frac{\hat\beta^{\hat\alpha} t^C}{C!\,\Gamma(\hat\alpha)} \lambda^{C+\hat\alpha-1} e^{-\lambda(t+\hat\beta)}.$$

As in the main text, we let $\hat\alpha' = C+\hat\alpha$ and $\hat\beta' = t+\hat\beta$.

$$\begin{aligned}P(\lambda|C,t,\hat\alpha,\hat\beta) &\propto \frac{\hat\beta^{\hat\alpha} t^C}{C!\,\Gamma(\hat\alpha)} \frac{\Gamma(\hat\alpha')}{\hat\beta'^{\hat\alpha'}} \left[\frac{\hat\beta'^{\hat\alpha'}}{\Gamma(\hat\alpha')} \lambda^{\hat\alpha'-1} e^{-\lambda(\hat\beta')}\right] \\ &\propto \frac{\hat\beta^{\hat\alpha} t^C}{C!\,\Gamma(\hat\alpha)} \frac{\Gamma(\hat\alpha')}{\hat\beta'^{\hat\alpha'}} \mathrm{DGamma}(\lambda;\hat\alpha',\hat\beta') \\ &\propto \mathrm{DGamma}(\lambda;\hat\alpha',\hat\beta'),\end{aligned}$$

hence these two probability densities are equal, justifying (2).

Supplementary Figures and Tables

	sequence count		
individual	raw	unique by well	unique overall
A	52,381,123	8,275,848	4,778,427
B	59,241,547	9,820,657	5,826,068
C	66,469,248	8,452,997	4,419,453

Table S1. Number of memory BCR sequences obtained by individual. "raw" refers to the number of reads obtained from sequencing, "unique by well" the number of unique sequences after performing clustering on reads for each barcoded PCR well, and "unique overall" the total number of unique sequences in the sample.

individual	cell type	in-frame	out-of-frame
A	naïve	0.93	0.92
	memory	0.08	0.15
B	naïve	0.91	0.89
	memory	0.22	0.27
C	naïve	0.92	0.90
	memory	0.20	0.28

Table S2. Fraction of BCR sequences that were identical to germline in the regions inferred to derive from germline. The fractions are stratified by individual, cell type, and frame status.

		Individual A				Individual B				Individual C			
		A	G	C	T	A	G	C	T	A	G	C	T
IGHV	germline	0.283	0.27	0.255	0.192	0.279	0.27	0.261	0.19	0.285	0.268	0.258	0.189
	sequence	0.277	0.261	0.256	0.206	0.276	0.266	0.261	0.197	0.282	0.265	0.258	0.196
IGHD	germline	0.199	0.328	0.141	0.332	0.196	0.323	0.157	0.324	0.197	0.326	0.153	0.324
	sequence	0.197	0.315	0.168	0.321	0.198	0.309	0.176	0.317	0.197	0.314	0.172	0.317
IGHJ	germline	0.197	0.428	0.22	0.154	0.2	0.424	0.223	0.154	0.186	0.438	0.225	0.151
	sequence	0.186	0.433	0.222	0.159	0.193	0.427	0.224	0.156	0.18	0.44	0.227	0.153

Table S3. Empirical stationary distribution for germline and observed sequences.

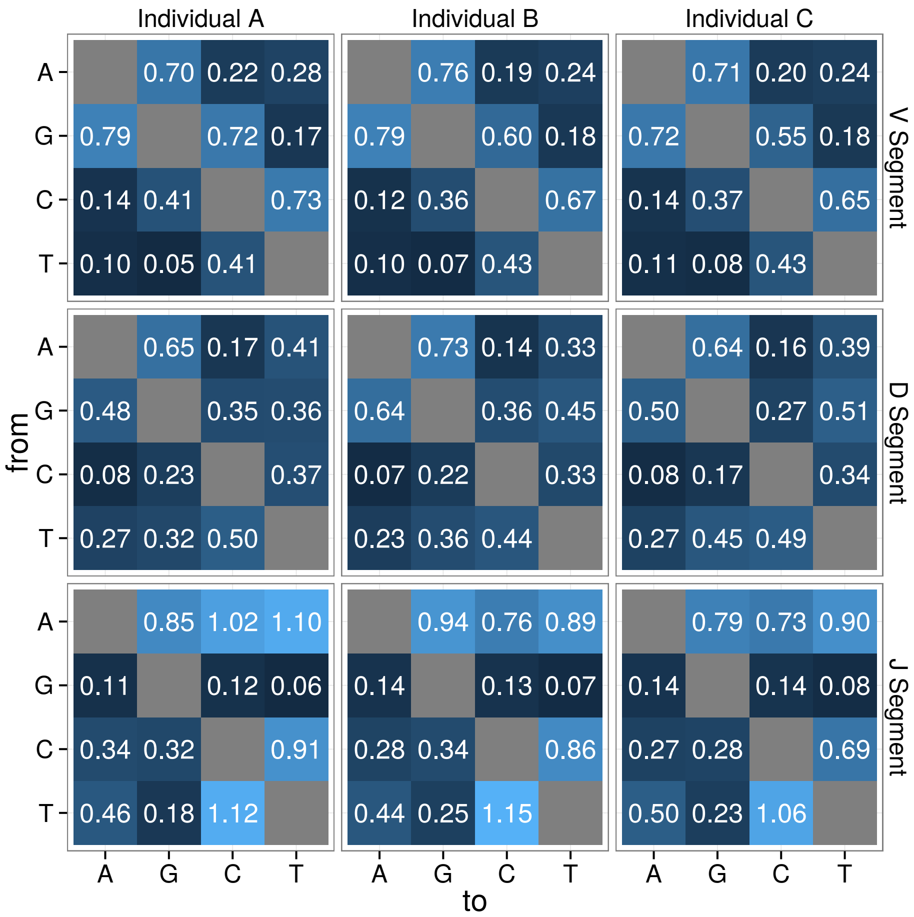

FIGURE S1. GTR coefficients for the $t_r\, Q_i\, \Gamma_i$ model estimated under maximum likelihood. Rows index the nucleotide found in the germline sequence, whereas columns index the nucleotide found in the observed sequence.

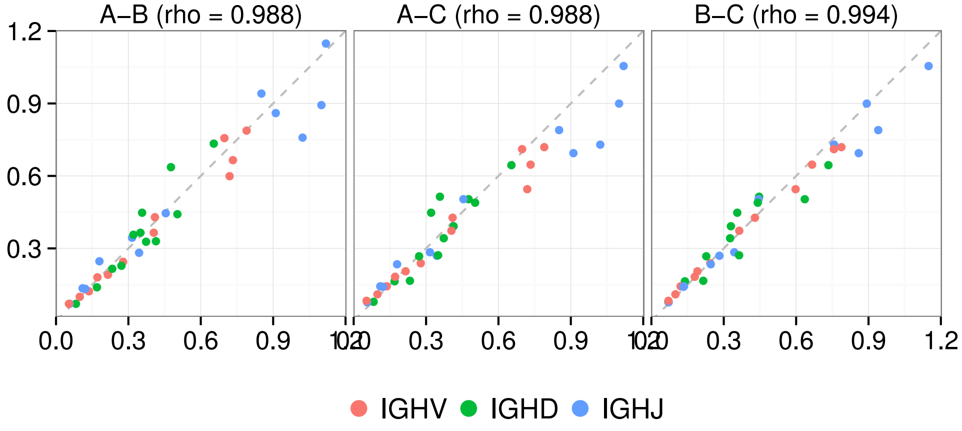

FIGURE S2. Pairwise comparison of off-diagonal entries in maximum-likelihood Q matrices under the $t_r Q_i \Gamma_i$ model between the three individuals. Coefficients are shown in Fig. S1.

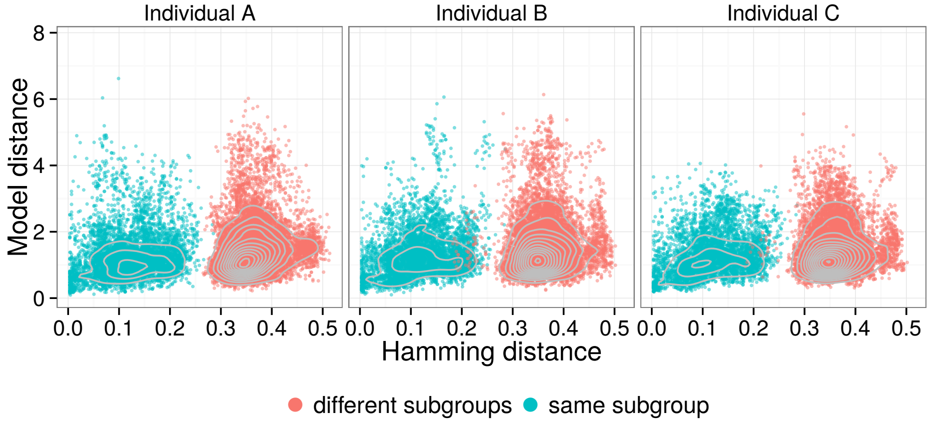

FIGURE S3. Comparison of Hamming distance between V genes (x-axis) and Euclidean distance between centered log-transformed median time transition matrices for productive rearrangements (y-axis). Colors indicate whether the V genes in a comparison come from the same or different subgroups. The correlation between the two was significant ($p < 10^{-15}$, Spearman's $\rho = 0.197$).

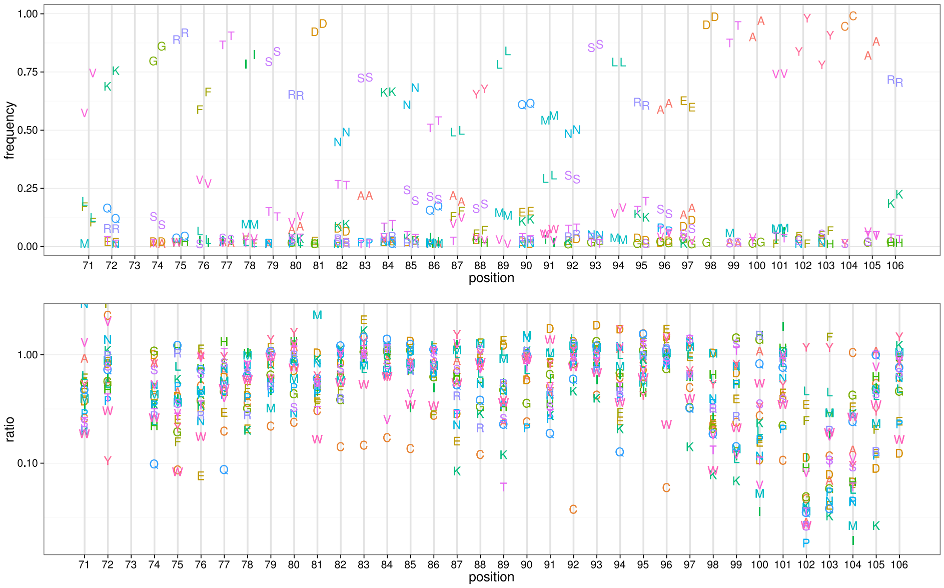

FIGURE S4. Amino acid profiles of out-of-frame and functional B cell sequences as aligned by the IMGT alignment. Top panel: frequency of amino acids per site. Letters to the left of the line show the profile for out-of-frame sequences and those to the right of the line show the profile for functional sequences. Bottom panel: amino acid frequency in functional sequences divided by that in out-of-frame sequences.

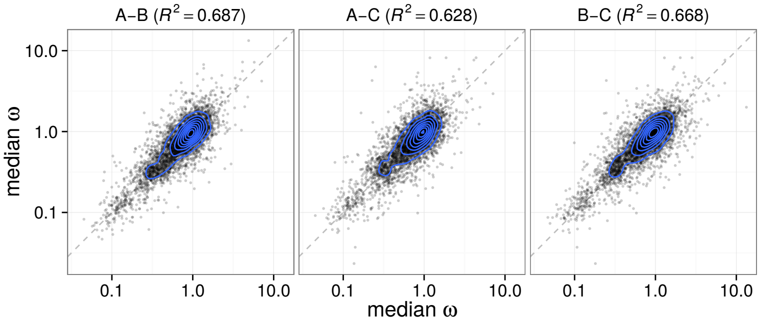

FIGURE S5. Pairwise comparisons of site-specific ω estimates between the three individuals along with the R^2 value from a linear model fit using $\log_{10}(\omega)$ for both the predictor (x-axis) and response (y-axis).

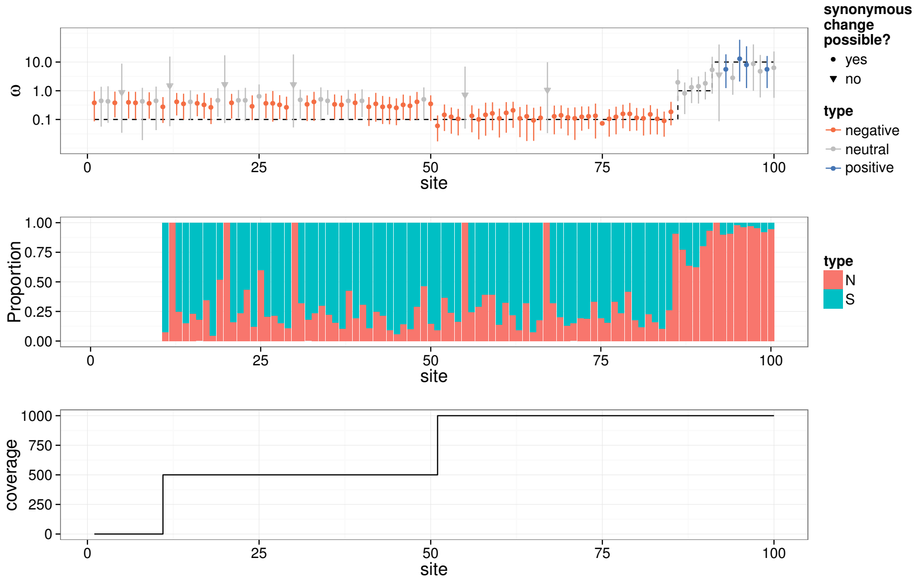

FIGURE S6. Top panel: site-specific ω estimates under simulated data with varying coverage. Inverted trianges show sites where the germline state was Tryptophan or Methionine, from which no synonymous changes are possible. Dashed black line shows simulated ω. Middle panel: proportion (second panel) of mutations at each position which were nonsynonymous (N) or synonymous (S). Bottom panel: sequence coverage by codon position.

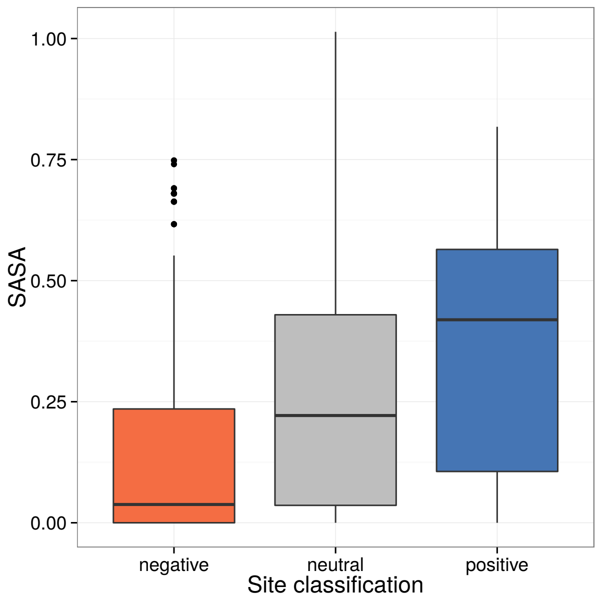

FIGURE S7. Normalized solvent-accessible surface area (SASA) values by per-site ω classification. A SASA value of 1.0 indicates that the residue is fully exposed, while a value of 0.0 indicates that the residue is buried. Sites under negative selection are significantly less exposed than sites under positive selection ($p < 10^{-12}$) or neutral selection ($p < 10^{-15}$) by Bonferroni-corrected Wilcoxon rank-sum test. Neutral sites were less exposed than sites under positive selection ($p < 0.002$).
